# Mitogen-Activated Protein Kinases Regulate Susceptibility to Ventilator-Induced Lung Injury

**DOI:** 10.1371/journal.pone.0001601

**Published:** 2008-02-13

**Authors:** Tamás Dolinay, Wei Wu, Naftali Kaminski, Emeka Ifedigbo, A. Murat Kaynar, Mária Szilasi, Simon C. Watkins, Stefan W. Ryter, Alexander Hoetzel, Augustine M. K. Choi

**Affiliations:** 1 Division of Pulmonary, Allergy and Critical Care Medicine, University of Pittsburgh, Pittsburgh, Pennsylvania, Unites States of America; 2 Department of Pulmonology, University of Debrecen Medical and Health Science Center, Debrecen, Hungary; 3 Department of Critical Care Medicine, University of Pittsburgh, Pittsburgh, Pennsylvania, United States of America; 4 Department of Cell Biology and Physiology, University of Pittsburgh, Pittsburgh, Pennsylvania, United States of America; 5 Department of Anesthesiology and Critical Care Medicine, University of Freiburg, Freiburg, Germany; 6 Division of Pulmonary and Critical Care Medicine, Brigham and Women's Hospital, Harvard Medical School, Boston, Massachusetts, United States of America; National Institutes of Health, United States of America

## Abstract

**Background:**

Mechanical ventilation causes ventilator-induced lung injury in animals and humans. Mitogen-activated protein kinases have been implicated in ventilator-induced lung injury though their functional significance remains incomplete. We characterize the role of p38 mitogen-activated protein kinase/mitogen activated protein kinase kinase-3 and c-Jun-NH_2_-terminal kinase-1 in ventilator-induced lung injury and investigate novel independent mechanisms contributing to lung injury during mechanical ventilation.

**Methodology and Principle Findings:**

C57/BL6 wild-type mice and mice genetically deleted for mitogen-activated protein kinase kinase-3 (*mkk-3* ^−/−^) or c-Jun-NH_2_-terminal kinase-1 (*jnk1* ^−/−^) were ventilated, and lung injury parameters were assessed. We demonstrate that *mkk3* ^−/−^ or *jnk1* ^−/−^ mice displayed significantly reduced inflammatory lung injury and apoptosis relative to wild-type mice. Since *jnk1^−/−^* mice were highly resistant to ventilator-induced lung injury, we performed comprehensive gene expression profiling of ventilated wild-type or *jnk1^−/−^* mice to identify novel candidate genes which may play critical roles in the pathogenesis of ventilator-induced lung injury. Microarray analysis revealed many novel genes differentially expressed by ventilation including matrix metalloproteinase-8 (MMP8) and *GADD45α*. Functional characterization of MMP8 revealed that *mmp8^−/−^* mice were sensitized to ventilator-induced lung injury with increased lung vascular permeability.

**Conclusions:**

We demonstrate that mitogen-activated protein kinase pathways mediate inflammatory lung injury during ventilator-induced lung injury. C-Jun-NH_2_-terminal kinase was also involved in alveolo-capillary leakage and edema formation, whereas MMP8 inhibited alveolo-capillary protein leakage.

## Introduction

Ventilator-associated lung injury arises as a clinical complication of mechanical ventilation. Its severe and advanced form, acute respiratory distress syndrome (ARDS), is associated with a high mortality and limited therapeutic options [1]–[3]. ARDS may contribute to multiple organ failure, a major cause of death in intensive care units [4]. Ventilated patients with otherwise healthy lungs seldom develop ventilator-associated lung-injury while those with pulmonary inflammation are predisposed to such injury [5], [6].

Animal models have been used extensively to model ventilator-induced lung injury (VILI) yet the underlying mechanisms remain incompletely understood. Recent research has focused on intracellular signaling pathways involved in the development of VILI, among which include the mitogen-activated protein kinase (MAPK) pathways, key regulators of inflammation [7]–[9]. MAPKs belong to an evolutionarily conserved and ubiquitous signal transduction superfamily of Ser/Thr protein kinases that regulate multiple cellular processes including apoptosis, growth, differentiation and responses to environmental stimuli. The MAPK superfamily includes three primary signaling cascades: the extracellular signal regulated kinases (ERK1/2), the c-Jun NH_2_-terminal kinases (JNK) and the p38 MAPKs. MAPK activation is associated with various forms of inflammatory lung injury. Therefore, strategies to modulate MAPK activation may have therapeutic benefit in this context [10], [11].

The global gene expression profiling approach has provided new insights into the mechanism of VILI. The observed differential activation of genes involved in the coagulation cascade, extracellular matrix production and intercellular communication in the context of VILI, suggests that this disease represents a complex rather than purely inflammatory process, where cellular mechanotransduction plays a key role [5], [12], [13].

The goals of this study were three fold: First we investigated the role of the p38 MAPK/MAPK kinase-3 (MKK3) and JNK signaling pathways in VILI. We measured lung injury parameters in response to ventilation in C57/BL6 (wild-type) mice and strains genetically deficient in MKK3 and JNK1. Second, we have assessed global gene expression changes in our model using microarray-based gene expression profiling. We describe series of genes differentially regulated by ventilation in either wild-type or *jnk1* ^−/− ^mice, and thereby provide insight into potential mechanisms of resistance to VILI. Third, we identify a role for matrix metalloproteinase-8 (MMP8) in alveolo-capillary permeability independent of MAPK.

## Materials and Methods

### Experimental design

Adult C57/BL6 (wild-type) mice and *mkk3, jnk1*, *mmp8* genetically deficient genotypes (−/−) were used for experiments (n = 192, weight = 20–30 g). Wild-type mice were purchased from Jackson Laboratory. *Mkk3^−/−^* and *jnk1^−/−^* mice were generated by R. Flavell (Yale University) and *mmp8^−/−^* mice were generated by S. Shapiro (University of Pittsburgh). All wild-type and genetically deficient mice used in this study were in the C57/BL6 background and matched for age and sex in all experiments. Mice were allowed to acclimate for 1 week with rodent chow and water ad libitum prior to the experiments. All animals were housed in accordance with guidelines from the American Association for Laboratory Animal Care. The Animal Care and Use Committee of the University of Pittsburgh approved the protocols. Mice were anesthetized with the intraperitoneal (i.p.) injection of a mixture of ketamine (150 mg/kg) and acepromazine (2.5 mg/kg) (Sigma-Aldrich Biochemical Co.). Tracheostomy was performed and a 20 G canula was inserted in the trachea. Groups of wild-type, *mkk3^−/−^* and *jnk1^−/−^* mice were randomized into 4 treatment conditions: control, 2-hour ventilation with 20 ml/kg tidal volume, 4-hour ventilation with 20 ml/kg tidal volume and 8-hour ventilation with 10 ml/kg tidal volume. Mice deficient in *mmp8^−/−^* were used for control and 8 hours mechanical ventilation conditions. Control animals were sacrificed immediately after anesthesia (n = 5–8 animals/group). The other animals were mechanically ventilated (n = 5–9 animals/group/condition) with room air using a Voltek RL-6 ventilator (Voltek Enterprises, Inc.). The ventilator setting included 2 cmH_2_O positive end-expiratory pressure (PEEP) and the lungs were recruited by inflation with up to 20 cmH_2_O pressures every hour. To relax chest muscles we used an hourly injection of 1 mg/kg i.p. of pancuronium bromide (Sigma-Aldrich). Animals were sacrificed at the end of the experiment with an overdose of ketamine (300 mg/kg). A detailed description of the experimental protocol is shown in Figure S1 of the online supporting information.

### Necropsy protocol, tissue and bronchoalveolar lavage fluid analysis

At the end of the experiment the abdomen and the chest of the animals was opened up. The left lung was isolated with surgical silk tied around the left main bronchus. The right lungs were lavaged using 0.5 ml saline (n = 5 animals/group/condition). The lavage volumes were inserted and withdrawn 3 times via the trachea canula to equalize volumes. 0.3–0.4 ml bronchoalveolar lavage fluid (BALF) was recovered per animal. One hundred microliter of BALF was mounted on glass slides by cytospinning for 10 minutes at 1000 rpm (Cytospin 3, Shandon Scientific) and stained with hematoxilin-eosin for differential cell count. Two hundred cells were counted per sample. The rest of the fluid was immediately centrifuged (2500 rpm for 10 minutes) at 4°C. The supernatant was stored at –20°C for cytokine analysis. The pellet was resuspended in 1 ml PBS for quantitative cell count. The cells were counted in a hemocytometer (Hausser Scientific). The right lung tissue was snap frozen and used for protein extraction. Subsequently, the ligature from the left main stem bronchus was removed and the lungs were used for histology. For microscopic morphology analysis, lungs were inflated with 0.5 ml of 2% paraformaldehyde through the tracheostomy canula for 1 minute then harvested (n = 5 animals/group). The lungs were fixed for 2 hours in 2% paraformaldehyde and submerged in 30% sucrose overnight. The following day, the tissue was snap frozen in liquid nitrogen and stored at –80°C. The frozen tissues were embedded and stained with hematoxylin-eosin for histology or used for cell death analysis (TUNEL staining). An experienced pathologist analyzed the lungs in a double-blinded fashion.

In a separate set of experiments, control and 8-hour mechanically ventilated lungs were harvested from wild-type and *jnk1* ^−/−^ mice and used for RNA extraction and immunohistochemistry (n = 4 samples/group). The right lung tissue was flash frozen and used for RNA extraction. The left lungs were fixed and used for immunohistochemistry.

### Lung microvascular permeability analysis and wet-to-dry weight ratio measurement

Evans Blue (EB) dye extravasation to the lung interstitium was used to quantify permeability changes following mechanical ventilation. EB dye (20 mg/kg, Sigma-Aldrich) was injected into the jugular vein of ventilated and control animals (n = 3–5 animals/group/condition). Mice were sacrificed 2 hours after dye injection [14]. Following the right main stem bronchus was tied off including the surrounding vasculature. The heart was punctured and 0.5 ml blood was collected. Blood was centrifuged to obtain plasma. The lungs were perfused with 1 ml normal 0.9% saline via the beating right ventricle to remove the remaining blood from the pulmonary circulation. The right lungs were harvested, blotted dry on paper towel and used for wet-to-dry weight ratio measurement. The left lungs were used for permeability measurement. Left lungs were homogenized in 1 ml 0.9% normal saline. For EB dye extraction and permeability analysis we used the method described by Belperio *et al* [15] with minor modifications. In brief, lung homogenates were incubated with formamide for at 60C for 12 hours and centrifuged at 2000 g for 30 minutes. EB dye in the plasma and in the lung tissue was quantified using a dual-wavelength spectrophotometer (620 nm and 740 nm). The following formula was used to correct for heme contamination at 620 nm absorbance (A): A_620_corrected = A_620_observed−(1.426×A_720_observed+0.03). We also used the universal turbidity correction factor for lung tissue described by Moitra *et al* [14]. We calculated the permeability index previously used by Belperio *at al.* [15] to quantify EB extravasation to the lung parenchyma using the following formula: (lung tissue A_620_corrected/g lung tissue)/plasmaA_620._

Lung wet-to-dry weight ratios were measured to quantify pulmonary edema formation. Harvested right lungs were blotted dry on paper towel and their weights were measured. Following lungs were desiccated in 60°C-heated oven over 5 days period to obtain a stabile dry weight [16]. Dried lung weights were measured and wet-to-dry ratio was calculated.

### Blood pressure experiments

Tail vein mean blood pressure was continuously monitored for wild-type mice receiving 8 hours 10 ml/kg mechanical ventilation (n = 3 animals) using non-invasive blood pressure cuffs (Columbus NIBP-1, Columbus Instruments).

### Cellular assays (ELISA, western blots)

The cytokine analysis of BALF for tumor necrosis factor-α (TNF-α) was carried out as described, using mouse specific kits (R&D Systems, Inc.) [17]. Total protein concentration was determined with the Coomassie Plus 200 Protein Assay (Pierce Biotechnology, Inc.). Western blot analysis was carried out as previously described for phosphorylated and dephosphorylated (total) forms of p38 MAPK and JNK [18], [19]. We used p38 MAPK Kinase Assay Kit for phospho-p38 MAPK detection. Total p38 MAPK expression was assessed using rabbit anti-mouse polyclonal antibody (Cell Signaling Technology, Inc). The phosphorylated and total forms of JNK MAPK were detected with rabbit anti-mouse polyclonal antibodies (Cell Signaling). We observed the expression of 3 forms of MMP8: pro-MMP8, active MMP8 and an inactive degradation product of MMP8 (85, 65 and 30 kDa molecular weight respectively) in lung tissue. MMP8 protein expression analysis was previously described by Tester *et al.* [20]. We used rabbit anti-mouse polyclonal MMP8 antibody for detection (Santa Cruz Biotechnology, Inc). GADD45α protein expression was assessed by Western immunoblotting using rabbit anti-mouse polyclonal antibody (Santa Cruz). GADD45α protein expression analysis was previously described by Fayolle et al [21]. β-actin expression was used as a loading control. Densities were quantified based on 3 individual samples per condition.

### Microarray analysis

Total RNAs were extracted from lung tissues with Trizol (Invitrogen Corp.) [22]. Complementary RNAs labeled with the Cy5 fluorescent dye was generated and hybridized to Codelink Uniset I 20K Bioarrays as recommended by the manufacturer (GE Healthcare Bio-Sciences Corp.) and previously reported by us [22]. All microarray data were normalized using the CyclicLoess normalization method (available at www.bioconductor.com) to remove noise in the data [23]. For analysis we filtered out genes that did not pass manufacturer recommendation for quality control. The log2-transformed expression data were used in the downstream analyses. We analyzed the gene expression patterns of 3 controls and 4 ventilation-treated animals. For statistical analysis we applied Welch's two-sample *t*-tests and non-parametric Threshold Number of Misclassification (TNoM) score from the Scoregene Package (available at http://compbio.cs.huji.ac.il/scoregenes) to find genes with significant expression changes. The *p*-values from the t-tests were adjusted for multiple testing using the false discovery rate (FDR) controlling procedure of Benjamini and Hochberg [24]. Genes which have the adjusted p-values less than 0.05, the TNoM scores equal to 0, and a fold-change in expression greater than 2 (on original data scale) were considered significantly changed [25]. This criterion for detecting differentially expressed genes is stringent, and can ensure the low false discovery rate. The color-coded heatmap was generated from significant genes using Java Treeview (available at www.sourceforge.net).

In order to find functional groups of genes over-represented in our dataset, we selected a bigger pool of differentially expressed genes using a less stringent criterion: p-values from t-tests (without FDR correction) less than 0.05, TNoM score equal to 0, and the fold-change in expression greater than 1.5 (on original data scale). The differentially expressed genes were analyzed using the GOstat program (available at www.gostat.wehi.edu.au), which identifies enriched functional groups of genes based on Gene-Ontology (GO) annotations. Gene groups with p-value<0.01 were considered overrepresented in our dataset (after FDR (p-value<0.05) correction). The data discussed in this publication have been deposited in NCBÌs Gene Expression Omnibus (GEO, http://www.ncbi.nlm.nih.gov/geo/) and are accessible through GEO Series accession number GSE7742.

### Real time TaqMan PCR

Quantitative RT-PCR was performed for 4 genes with increased expression following mechanical ventilation in wild-type and *jnk^−/−^* mice (n = 4 samples/condition). TaqMan PCR was executed as described previously [22] . Commercially available Assay-on-Demand primer probe sets (Applied Biosystems, Inc.) were used for Gadd45α (Mm00432802_m1), for Wisp2 (Mm01247817_m1) for Atf4 (Mm00515324_m1) and for Mmp8 (Mm00772335_m1). Gene expression was measured relative to an endogenous reference gene, mouse β-glucuronidase (Gusb Mm00446957_m1). The results were log_2 _base transformed and the arithmetical means of 4 measurements were compared.

### Tissue immunohistochemical-staining and terminal transferase-mediated dUTP nick-end labeling assay

Lung tissues were fixed with 2% paraformaldehyde, merged in 30% sucrose overnight and snap frozen. Sections of lung were prepared and immunostaining was performed as described [26]. We used GADD45α rabbit polyclonal primary antibody (Chemicon International, Inc.) and goat polyclonal IgG primary antibody for MMP8 (Santa Cruz Biotechnology, Inc.). For terminal transferase-mediated dUTP nick-end labeling (TUNEL) assay FITC-Avidin D dye was used [27]. Results were quantified based on 4 individual samples/group.

### Statistics

Results are presented mean±SEM. Kruskal-Wallis test was performed for multiple group comparison and intergroup differences were analyzed with Wilcoxon Rank Sum Test [28] with SPSS statistics software (SPSS, Inc.). Significance level was p<0.05. Microarray statistics and tools are detailed above.

## Results

### Mechanical ventilation induces lung injury in vivo

**Figure 1:**
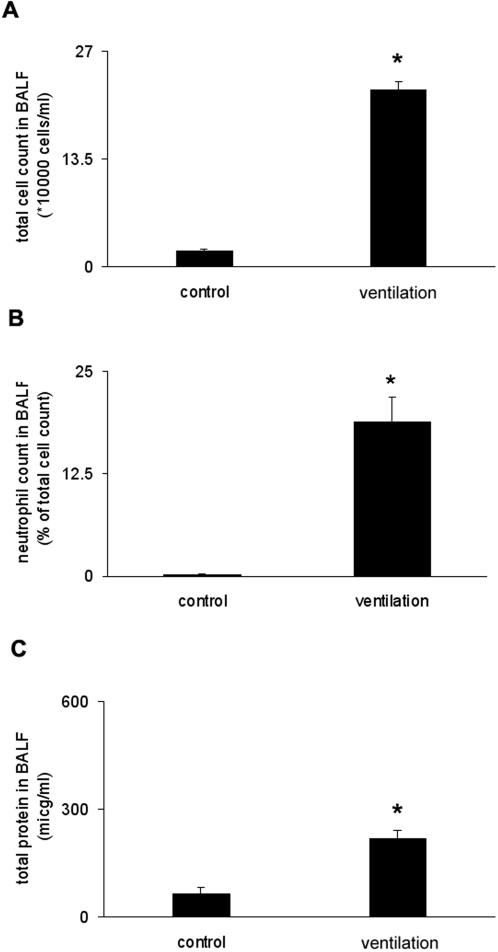
Effects of ventilation on indirect indices of injury in the BALF. (A) Mechanical ventilation (10 ml/kg, 8 hours) resulted in increased cell count in BALF. (B) Lavage neutrophil cell count increased following ventilation (% of total cell count). (C) Ventilation led to significant total protein increase in the BALF. *represents significant differences between ventilated and control animals, p<0.05. Control = no ventilation.

We ventilated C57/BL6 mice using moderate tidal volume ventilation (10 ml/kg, 8 hours) with low PEEP (2 cmH_2_O). This ventilation strategy allowed us to observe neutrophil influx into the lungs without significant animal mortality and resulted in substantial lung injury. For all animals, total cell count and total protein content were measured in the bronchoalveolar lavage fluid (BALF). Mechanical ventilation resulted in the increase of total cell count in the BALF (Figure 1A), and significant neutrophil recruitment to the alveoli (Figure 1B). We also observed increased protein content in the BALF following 8 hours mechanical ventilation (Figure 1C).

**Figure 2:**
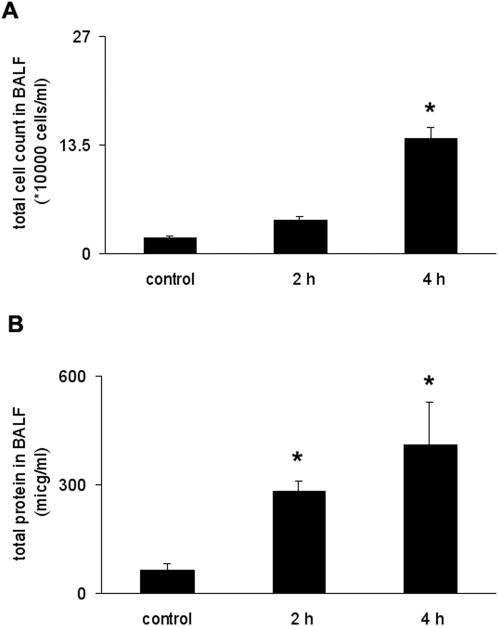
Time course of mechanical ventilation-induced lung injury with high tidal volume. (A) Total cell count increased following ventilation (20 ml/kg) for 2 and 4 hours (*10000 cells/ml). (B) BALF total protein levels (microgram/ml) were augmented by mechanical ventilation. *represents significant differences between ventilated and control animals, p<0.05.

**Figure 3:**
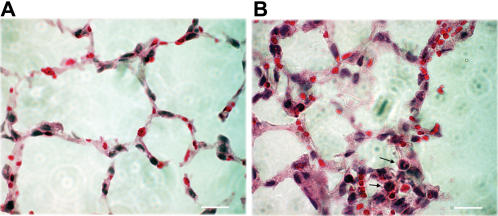
Mechanical ventilation-induced neutrophil leukocyte infiltration in lung tissue. (A) Control (non-ventilated) lung tissue. (B) Mechanical ventilation-induced lung injury following mechanical ventilation (10 ml/kg, 8 hours). Arrows point at mononuclear leukocytes in the alveoli. Hematoxylin-eosin staining, 40-fold magnification, scalebar = 50 micrometer. A representative picture is shown.

To maximize lung injury following mechanical ventilation mice were ventilated with high tidal volume (20 ml/kg) and low PEEP (2 cmH_2_O) for 2–4 hours. High tidal volume ventilation was used to induce rapid changes in cell count, alveolo-capillary permeability and MAPK activation. Total cell count in the BALF increased in a time-dependent fashion during the course of mechanical ventilation (20 ml/kg), (Figure 2A). Total protein level in the BALF was significantly higher as early as 2 hours of ventilation when compared to controls (Figure 2B). The levels of the pro-inflammatory cytokine tumor necrosis factor-alpha (TNF-α) were also analyzed in the BALF. TNF-α levels were increased after 2 hours of ventilation (*data not shown*), but were not detectable at later ventilation time (4–8 hours). The detailed description of qualitative cell count and total protein is shown in Table S1. Hematoxylin-eosin stained lung tissues were compared following mechanical ventilation. Mechanical ventilation resulted in lung tissue injury marked by alveolar-septal thickening and neutophil granulocyte infiltration to the parenchyma (Figure 3). Mean blood pressure was continuously monitored during mechanical ventilation and significant blood pressure drop was noticed only during the 8^th^ hour (Table S2).

### Mice deficient in MKK3 and JNK1 are less susceptible to ventilator-induced lung injury

**Figure 4:**
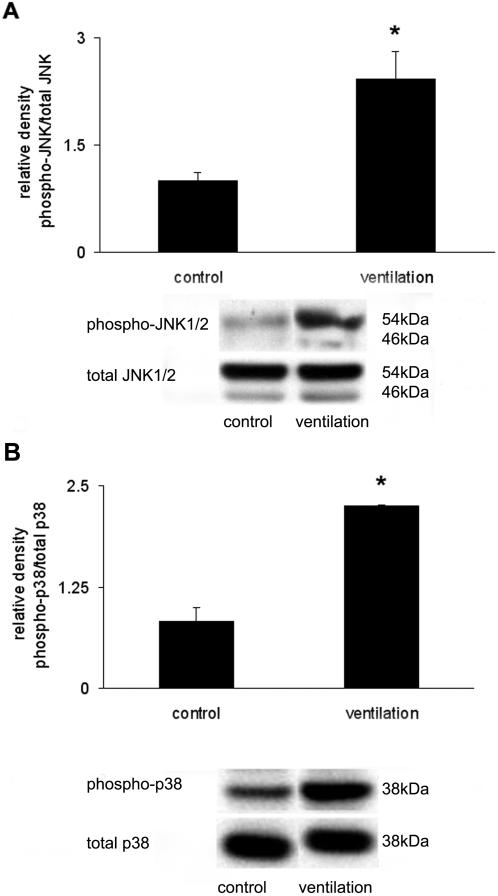
JNK and p38 MAPK protein activation increased following ventilation. (A) Quantified c-Jun-NH_2_-terminal kinase (JNK) activation data, expressed as phospho/dephospho (total) JNK following mechanical ventilation (20 ml/kg, 4 hours). (B) Increased p38 mitogen-activated protein kinase (p38 MAPK) activation was also detected after 4 hours ventilation and expressed as phospho/total p38. Representative blots for phosphorylated and total forms of proteins are shown. *represents significant differences between ventilation and control animals.

**Figure 5:**
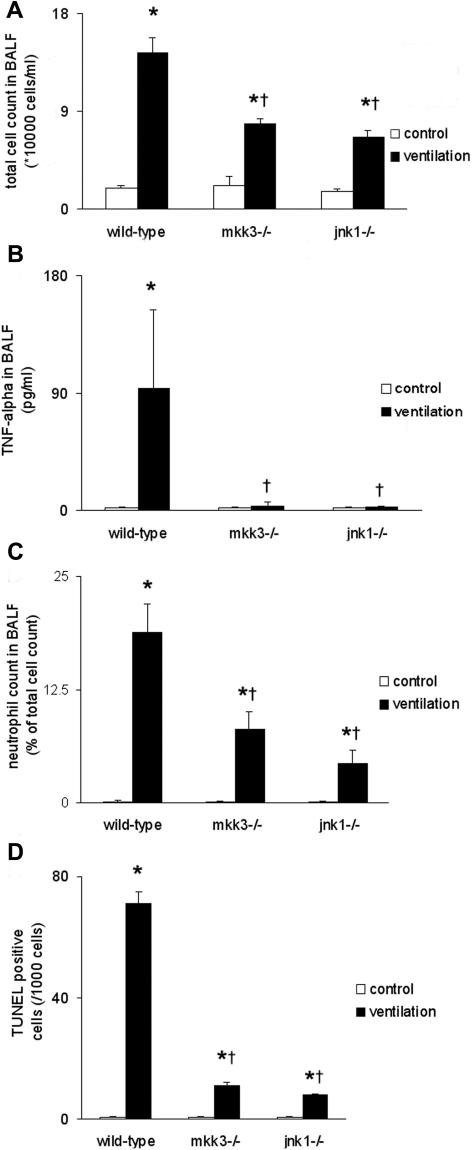
Lung injury and cell death measurement in wild-type, *mkk3* ^−/− ^and *jnk1* ^−/−^ mice. (A) Reduced total cell count was detected in genetically deficient mice following ventilation (20 ml/kg, 4 hours). (B) Tumor necrosis factor-α levels were reduced in genetically deficient mice after ventilation (20 ml/kg, 2 hours). (C) Neutrophil levels were lower in genetically deficient mice following ventilation (10 ml/kg, 8 hours). (D) Quantified TUNEL staining for cell death showed that wild-type mice have increased cell death when compared to *mkk3* ^−/− ^and *jnk1* ^−/− ^mice following volume ventilation (10 ml/kg, 8 hours). Values expressed as number of TUNEL staining cells/1000 cells. *represents significant differences between control and ventilated animals, † represents significant differences between wild-type and genetically deficient animals.

We examined whether VILI affects the activation of JNK1 and p38 MAPK, members of major inflammatory signaling pathways involved in lung injury. The levels of phosphorylated JNK and p38 MAPK were increased following mechanical ventilation (20 ml/kg, 4 hours), as determined by Western immunoblot analyses of corresponding phospho- and dephospho- forms (Figure 4A and B). To further assess the role of p38 MAPK and JNK1 in VILI, we ventilated mice genetically deficient either in *mkk3* (*mkk3* ^−/−^), the major upstream activating kinase of p38 MAPK, or *jnk1* (*jnk1* ^−/−^), and compared their lung injury indices to that of wild-type mice. The *mkk3* ^−/− ^mice were used for study since p38 MAPK deficient mice are embryonic lethal. In the absence of ventilation, no differences between the three strains were observed with respect to total cell count, protein, or TNF-α levels in the BALF. Mechanical ventilation (20 ml/kg, 4 hours) resulted in significantly higher total cell count in the BALF of wild-type mice relative to that of *mkk3* ^−/− ^or *jnk1* ^−/−^ mice (Figure 5A). TNF-α levels were significantly higher in wild-type animals than in *mkk3^−/−^* and *jnk1^−/−^* mice (Figure 5B). TNFα analysis was performed after 2 hours ventilation due to the early and transient nature of this response.

When neutrophil influx was quantified following ventilation (10 ml/kg, 8 hours), we found that both *mkk3* ^−/− ^and *jnk1* ^−/−^ mice exhibited reduced leukocyte infiltration when compared to wild-type mice (Figure 5C). Lung cell death was also assessed with TUNEL staining and we observed that wild-type mice showed significantly higher numbers of positively staining cells, compared to *mkk3* ^−/− ^and *jnk1* ^−/−^ mice (Figure 5D). To localize cellular death in the lung tissue we performed cell specific immunostaining. TUNEL staining was principally localized to the alveolar epithelium. Other cell stains using neutrophil granulocyte marker (Ly6), macrophage (Mac1) and endothelial cell-specific (CD31) markers did not reveal increased apoptosis in these cell types (*data not shown*).

**Figure 6:**
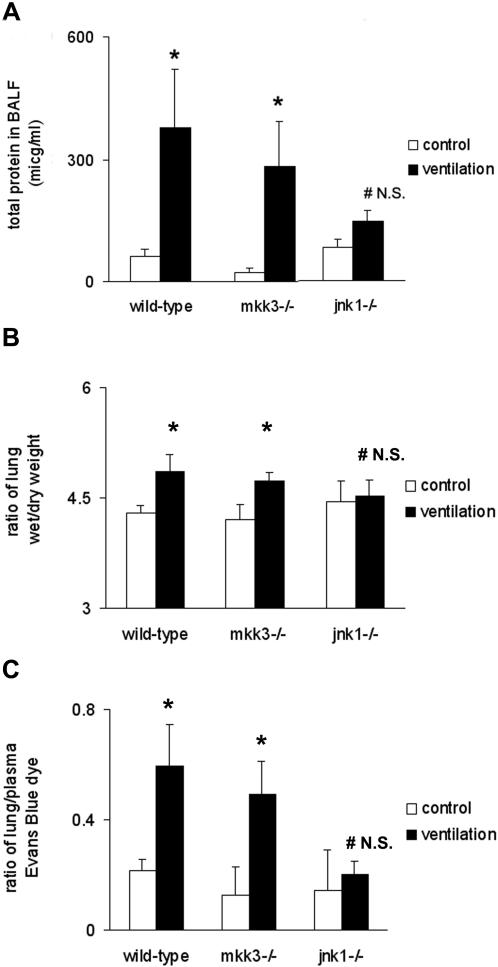
Alveolar protein content, lung microvascular permeability, and wet-to-dry weight ratio measurement in wild-type, *mkk3* ^−/− ^and *jnk1* ^−/−^ mice. (A) Total protein levels were significantly increased in wild-type and *mkk3* ^−/− ^mice following ventilation (20 ml/kg, 4 hours). No differences in total protein levels were observed in the *jnk1* ^−/− ^mice. (B) Wild-type and *mkk3* ^−/−^ mice responded to mechanical ventilation with pulmonary edema formation (20 ml/kg, 4 hours). Pulmonary edema formation was quantified by measuring the wet and desiccated dry weight of lung tissue. No significant lung water changes were detected in *jnk1* ^−/−^ mice. (C) Increased microvascular permeability was detected with Evans Blue (EB) dye extravasation method in wild-type and *mkk3* ^−/−^ mice following mechanical ventilation (20 ml/kg, 4 hours). Mice lacking *jnk1* gene did not show increased permeability following mechanical ventilation. Changes in permeability are expressed as a ratio of lung tissue/blood plasma to correct for differences in volumes of injected dye to the circulation.. *represents significant differences between control and ventilated animals. ^#^ N.S. = non-significant change between control and ventilated mice.

Wild-type or *mkk3* ^−/−^ mice responded to mechanical ventilation with significant increase of BALF protein levels relative to their corresponding non-ventilated controls. However, ventilation did not affect the protein levels in *jnk1* ^−/−^ mice (Figure 6A). Subsequently, we assessed pulmonary edema and alveolo-capillary permeability changes in mechanically-ventilated mouse strains. Wet-to-dry lung weight ratios indicated increased lung water content in wild-type and *mkk3* ^−/−^ mice, but not in *jnk1* ^−/−^ mice which were resistant to pulmonary edema formation (Figure 6B). Analysis of Evans Blue dye extravasation revealed increased microvascular permeability in both wild-type and *mkk3* ^−/−^ mice (Figure 6C). However no permeability changes were observed in *jnk1* ^−/−^ mice following 4 hours mechanical ventilation (Figure 6C). When total protein levels were assessed in the BALF at 8 hours we found that there was a modest increase in *jnk1* ^−/−^ mice as well (Table S1). These findings further support the observation that mice lacking JNK1 or MKK3 are less susceptible to ventilator-induced inflammation and cell death. Additionally, we found that *jnk1* ^−/−^ mice are also relatively resistant to pulmonary edema formation.

### Differential expression of genes in VILI

**Figure 7:**
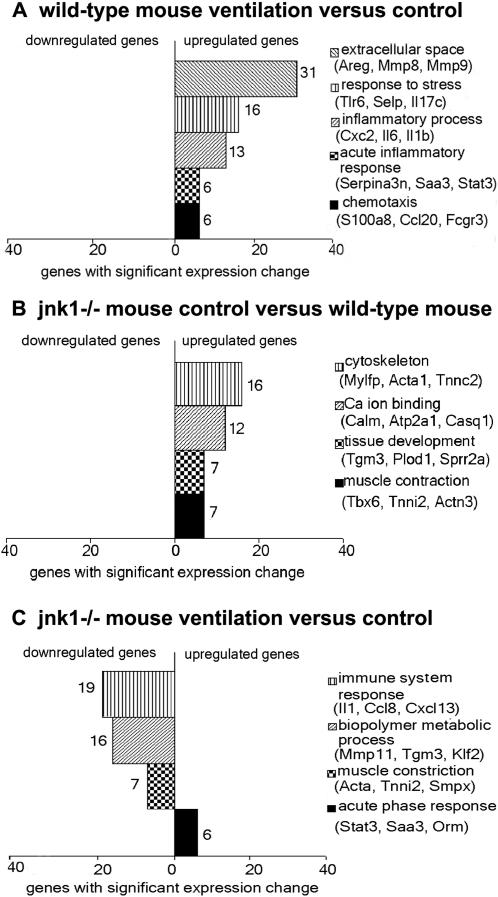
Gene Ontology analysis. Gene expression ratios were analyzed under the following conditions: (A) wild-type mouse ventilation versus control (total genes: 335), (B) *jnk1^−/−^* mouse control versus wild-type mouse control (total genes: 314), and (C) *jnk1* ^−/− ^mouse ventilation versus control (total genes: 686). Values represent gene numbers enriched in a functional annotation. Functional groups of upregulated genes are shown on the right side of the panel and groups of downregulated genes are shown on the left side of the panel. Gene symbols and names: amphiregulin (Areg), matrix metalloproteinase-8 and 9 (Mmp8, Mmp9), toll-like receptor 6 (Tlr6), p-selectin (Selp), interleukin 17C (Il17c), chemokine ligand 2 (Cxcl2), interleukin 6 and 1b (Il6, Il1b), serine protease 3 (serpina3), serum amyloid A3 (Saa3), signal transducer and activator of transcription 3 (Stat3), calgranulin A (S100a8), chemokine ligand 20 (Ccl20), IgG Fc receptor (Fcgr3), myosin-light chain (Myflp), alpha1 actin (Acta1), troponin C2 (Tnnc2), calmodulin (Calm), atpase 2A1 (Atp2a1), calsequestrin 1 (Casq1), transglutaminase 3 (Tgm3), lysine hydroxylase 1 (Plod1), small proline-rich protein 2A (Sprr2a), t-box 6 (Tbx6), troponin I2 (Tnni2), alpha3 actinin (Actn3), interleukin 1 (Il1), monocyte chemotactic protein 2 (Ccl8), chemokine receptor 13 (Cxcl13), eg. matrix metalloproteinase 11 (Mmp11), kruppel-like factor 2 (Klf2),alpha actin (Acta), troponin I2 (Tnni2), small muscle protein (Smpx), orosomucoid (Orm).

**Figure 8:**
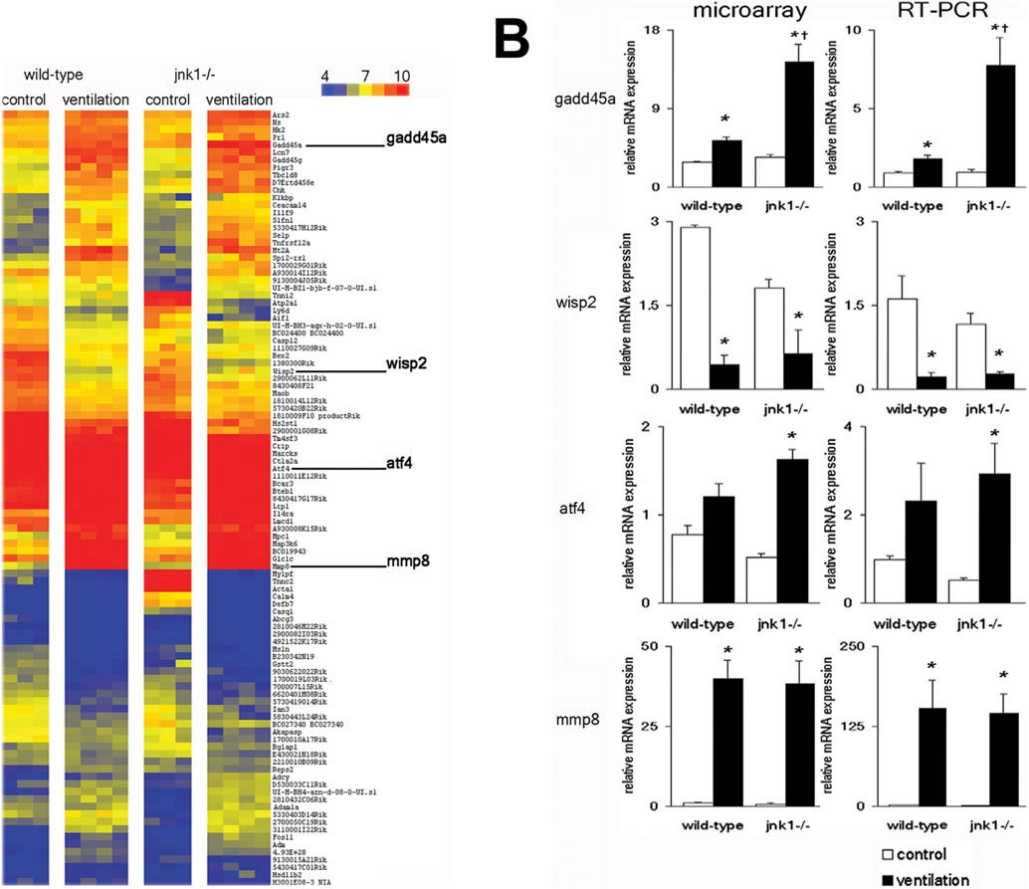
Differential gene expression change following mechanical ventilation in wild-type and *jnk1^−/−^* mice. (A) The pattern of 103 differentially expressed genes is displayed. The gene expression profile of controls and animals subjected to mechanical ventilation (10 ml/kg, 8 hours) is compared. Confirmed genes: growth arrest and DNA-damage-inducible protein-45alpha (*gadd45α*), WNT1 inducible signaling pathway protein 2 (*wisp2*), activating transcription factor 4 (*atf4*) and *mmp8* are highlighted. red = upregulation, blue = downregulaton, yellow = null expression. Big columns represent wild-type control, wild-type ventilated, *jnk1^−/−^* control and *jnk1^−/−^* ventilated mice. Small columns show the gene expression profile of one animal. Each row represents one gene. Gene symbols are shown on right side of the panel. For complete gene names please refer to Table S3. (B) Microarray and confirmatory TaqMan RT-PCR expression profile of *gadd45α*, *wisp2*, *atf4* and *mmp8* genes. The increased expression *gadd45α* following ventilation was identified by microarray and verified with RT-PCR. *Jnk1* ^−/−^ mice had a higher expression of *gadd45α* after ventilation than wild-type mice. *Wisp2* expression was decreased in both wild-type and *jnk1* ^−/− ^mice following mechanical ventilation. RT-PCR confirmed the microarray findings. Microarray results showed that *atf4* gene was regulated only in *jnk1* ^−/− ^mice and RT-PCR verified this finding. *Mmp8* showed increased expression with ventilation on microarray and with RT-PCR verification, which was similar in both wild-type and *jnk1* ^−/− ^mice. *represents significant differences between control and ventilated animals, †represents significant difference between wild-type ventilated and *jnk1^−/−^* ventilated mice.

To assess possible differences in gene expression patterns following mechanical ventilation, we used gene expression analysis by microarray. Since *jnk1* ^−/− ^mice were the most resistant to VILI we also compared the gene expression profile of *jnk1* ^−/− ^mice to wild-type mice in the absence or presence of ventilation. To identify groups of genes which are significantly overrepresented in our dataset, we used Gene Ontology (GO) analysis (t-test p-value<0.05, TNoM score = 0 and fold-change of expression >1.5, see “Materials and Methods” for details). Wild-type mice responded to mechanical ventilation with the significant enrichment (p<0.05) of upregulated genes in extracellular matrix, stress-response and inflammation-related functional groups (Figure 7A). In order to assess how *jnk1* ^−/− ^mice differ from wild-type mice we analyzed gene expression patterns prior to and following mechanical ventilation. When control (wild-type and *jnk1* ^−/−^) mice were compared in the absence of ventilation, *jnk1* ^−/− ^mice showed the significant enrichment of upregulated genes in cytoskeleton, calcium ion binding and muscle contraction functional groups (Figure 7B). Mechanical ventilation in *jnk1* ^−/−^ mice resulted in the significant enrichment of downregulated genes in immune system function, biopolymer metabolism and muscle contraction functional groups and the significant enrichment of upregulated genes in the acute phase response functional group (Figure 7C). These results strengthen our findings that *jnk1* ^−/−^ mice respond to lung injury with an altered inflammatory response. Using more stringent statistical criteria (t-tests p-value<0.05 with FDR correction, TNoM score = 0 and fold-change of expression >2, see “Materials and Methods” for details) we identified 103 genes whose expression significantly changed following ventilation in wild-type and/or in *jnk1* ^−/− ^mice. Expression of these genes is shown in a color-coded heatmap format in Figure 8A. Gene names with expression values are included in the online data supplement (Table S3). We confirmed the expression results of four differentially expressed genes (Figure 8B). Growth arrest and DNA-damage-inducible protein-45alpha (*gadd45α*), WNT1 inducible signaling pathway protein 2 (*wisp2*), activating transcription factor 4 (*atf4*) and matrix metalloproteinase-8 (*mmp8*) microarray expression findings corresponded with TaqMan RT-PCR results (Figure 8B).

**Figure 9:**
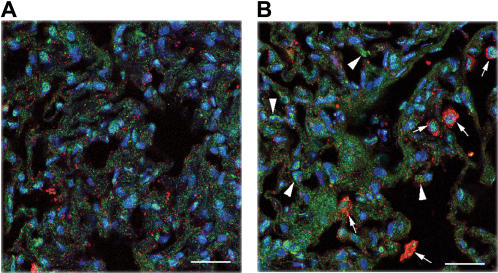
Immunohistochemistry dual staining for GADD45α and MMP8. Wild-type animals were stained for GADD45α and MMP8 prior to (A) and following mechanical ventilation (10 ml/kg, 8 hours), (B). Both gene products showed increased expression following ventilation. The staining pattern suggests cytosolic staining in multiple cell types for GADD45α and staining for MMP8 restricted to inflammatory cells at the cell membrane. Large arrowheads indicate GADD45α staining cells. Small arrows indicate MMP8 staining cells. Green stain = GADD45α, red stain = MMP8, blue stain = counter nuclear staining. 40-fold magnification, scale bar = 50 micrometer.

**Figure 10:**
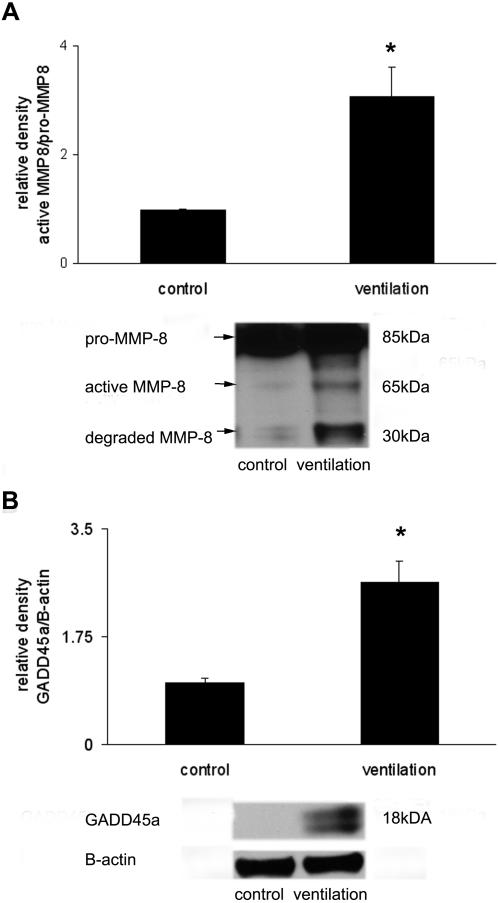
Mechanical ventilation induces MMP8 and GADD45α expression in mice. (A) Quantified MMP8 activation data, expressed as active/pro-MMP8 following mechanical ventilation (10 ml/kg, 8 hours). Arrows point at different forms of MMP8. (B) Increased GADD45α expression was also detected after 8 hours ventilation and expressed as GADD45α/loading control β-actin. Representative blots are shown. *represents significant differences between ventilation and control animals.

We chose to further validate the expression of *gadd45α* and *mmp8*. The *mmp8* gene was chosen because of its remarkable increase in expression following mechanical ventilation (Figure 8B). *Gadd45α* expression was increased following mechanical ventilation, and this increase was significantly higher in *jnk1^−/−^* mice when compared to wild-type mice (Figure 8B). Co-immunostaining was performed for GADD45α and MMP8 proteins in lung tissue from wild-type mice (Figure 9, A and B). Increased GADD45α staining was evident in ventilated wild-type mice relative to non-ventilated controls. Multiple cell types expressed GADD45α protein, which localized primarily in the cytoplasm. MMP8 expression was also increased following ventilation but it was restricted to certain cell types, presumably mononuclear cells, and localized primarily in the cell membrane (Figure 9, A and B). Immunoblot analysis showed increased protein expression of MMP8 (Figure 10A) and GADD45α (Figure 10B) following mechanical ventilation.

### MMP8 regulates microvascular permeability in lung injury

**Figure 11:**
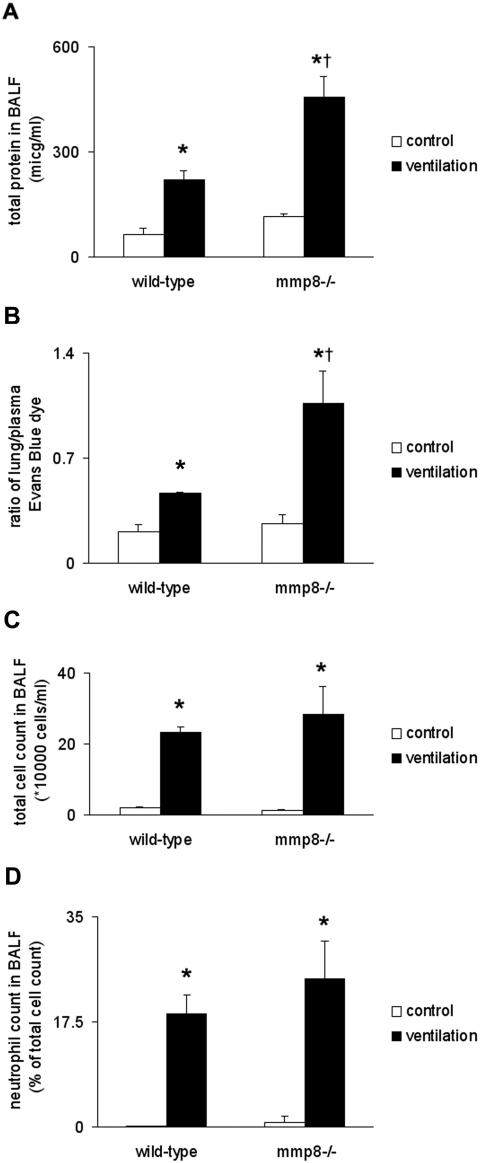
Indirect lung injury indices of mice deficient in *mmp8.* (A) Total protein levels were significantly higher in *mmp8* ^−/−^ mice than in wild-type mice following mechanical ventilation (10 ml/kg, 8 hours). (B) Microvascular permeability, assessed with EB dye extravasation was significantly higher in *mmp8* ^−/−^ mice than in wild-type mice following mechanical ventilation. (C) Total cell counts were not modified by MMP8. (D) MMP8 did not affect neutrophil recruitment to the lung at 8 hours. *represents significant differences between control and ventilated animals, † represents significant differences between wild-type and *mmp8* ^−/−^ mice.

To assess the physiological relevance of MMP8 in VILI, *mmp8* gene deleted mice (*mmp8* ^−/−^) were ventilated and their lung injury parameters were compared to wild-type mice. The *mmp8* ^−/− ^mice displayed increased total protein levels in the BALF following ventilation, relative to wild-type mice (Figure 11A). Increased Evans Blue dye extravasation was also detected in *mmp8* ^−/− ^mice when compared to wild-type mice (Figure 11B), whereas no differences in lung edema formation were observed (*data not shown*). However differences in total and differential cell count in the BALF were not detected between the two strains (Figure 11C and D).

## Discussion

### The role of mitogen-activated protein kinases in ventilator-induced lung injury

Although the pathology of VILI has attracted intensive clinical and experimental investigation, at present, the mechanisms of injury remain only partially explored [29]. MAPK are involved in transmitting various extracellular signals from the cell surface to the nuclei but have only recently been suggested as mediators of VILI [30]. Uhlig *et al.* described the increased activation of JNK, p38 and ERK MAPK following *in vivo* mechanical ventilation of rats for 1 hour. JNK and ERK1/2 activation were significantly larger when high-pressure ventilation (equal to 48 ml/kg tidal volume in their model) was used instead of low pressures (equal to 18 ml/kg tidal volume). p38 MAPK activation increased approximately 2 fold following 1-hour high pressure mechanical ventilation [30]. Increased activation of ERK1/2, JNK and p38 MAPK were also described by Li *et al.* in a mouse model of VILI [31]. In our setting we also detected increased JNK and p38 MAPK activation following mechanical ventilation. We were interested to explore how mice respond to mechanical ventilation in the absence of major MAPK signaling pathways. *Mkk3* ^−/−^ and *jnk1* ^−/−^ mice that were subjected to the same ventilation regimen as their wild-type counterparts were more resistant to VILI. Li *et al.* also reported altered neutrophil sequestration in the lung tissue when *jnk1* ^−/−^ and *jnk2* ^−/−^ mice were ventilated with high tidal volume (30 ml/kg) for 5 hours [31]. It is important to point out that in our model significant neutrophil influx was only detected after 8 hours, which may be a result of the lower tidal volumes used in this study. In addition to inflammation, we observed increased alveolar protein accumulation, lung microvascular permeability and pulmonary edema formation in our model of VILI that suggests increased alveolo-capillary barrier permeability. Double-isotope imaging studies of ventilated rats performed by de Prost *et al.* showed that mechanical ventilation with pressures greater than 20 cmH_2_O, (corresponding to 13.7±4.69 ml/kg tidal volume in their setting) results in permeability changes in both alveolar and capillary membranes [32]. In our model, pulmonary edema formation preceded neutrophil granulocyte infiltration to lung parenchyma during mechanical ventilation, suggesting that permeability changes can also take place without significant inflammation. Previously Quinn *et al.* reported similar findings in a rat model of VILI [33]. We observed only moderate blood pressure drop in our animals following 7 hours of mechanical ventilation and therefore cardiogenic edema formation is unlikely to be the reason for the early increase in lung water content. Our results show that mechanical ventilation resulted in increased alveolo-capillary permeability and subsequent pulmonary edema formation in *mkk3^−/−^* mice, whereas *jnk1^−/−^* mice were relatively resistant to alveolar fluid accumulation when compared to wild-type mice. Petrache *et al.* demonstrated that cultured human pulmonary endothelial cells respond to TNF-α stimulation with microtubule destabilization and subsequent barrier dysfunction [34]. The lack of TNF-α response could, at least partially, account for delayed increase in lavage protein levels in *jnk1^−/−^* mice. Another possible explanation for relative resistance to alveolar protein-rich fluid accumulation is reduced epithelial apoptosis in *jnk1^−/−^* mice. Increased apoptosis was described in animal models of VILI [35] and in human ARDS [36]. Multiple cell types were shown to undergo apoptosis in response to acute lung injury including alveolar epithelial cells, endothelial cells and polymorphonuclear granulocytes [37]–[39]. In our model TUNEL staining displayed an epithelial localization. Given the moderate tidal volume mechanical ventilation we argue that the observed cell death is not severe and it is due to apoptosis rather than epithelial cell necrosis. JNK MAPK pathways are involved in pro-apoptotic signaling and p38 MAPK isotypes can be pro- or anti-apoptotic [40], [41]. A reduced cell death response was observed in both *mkk3* ^−/−^ and *jnk*1^−/−^ mice, but the reason why only *jnk1^−/−^* mice showed reduced alveolo-capillary permeability remains to be investigated. Recently Li et *al.* also showed decreased edema formation and apoptosis in ventilated *jnk1^−/−^* mice when compared to their wild-type counterparts [42].

In our model both high and moderate tidal volume ventilation strategies with low PEEP resulted in lung injury. Previously Tremblay et *al.* [17] and Belperio et *al.* [15] showed that moderate tidal volume ventilation with low PEEP increases proinflammatory cytokine and neutrophil sequestration in rodent lungs. Our results show that extended moderate tidal volume ventilation with low PEEP can induce injury comparable to higher tidal volumes.

### Mechanisms of ventilator-induced lung injury

**Figure 12:**
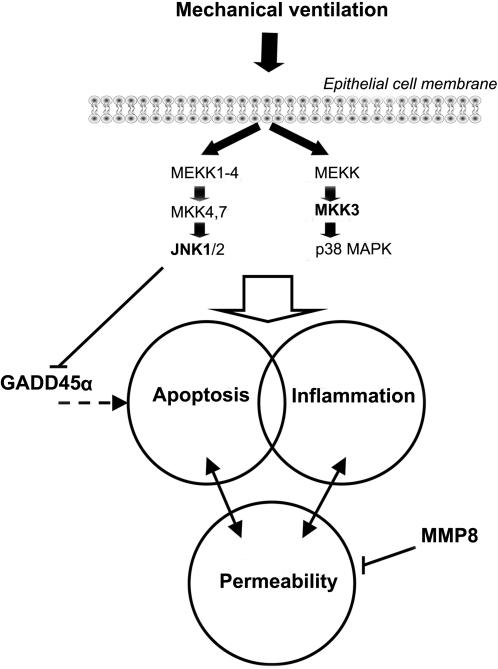
Theoretical interactions of MAPK, GADD45α and MMP8 in alveolar epithelial cells. The p38 and JNK MAPKs transmit injury signals from the cell surface to the nucleus resulting in inflammation and apoptosis. Alveolar-capillary permeability interacts with inflammatory and apoptotic pathways. JNK1 MAPK inhibits GADD45α expression, which effects apoptotic signaling. MMP8 protects against increased alveolo-capillary permeability. Dashed line = theoretical connection. Arrow = activation, bar = inhibition

A further aim of our experiments was to assess mechanisms of VILI. Expression changes of inflammation (eg., Il6, Il1b), stress response (eg., Tlr6, Selp) and extracellular matrix-related genes (eg., Areg, Mmp9) dominated in wild-type mice following 8 hours ventilation (Figure 7A). Similar gene expression patterns have been previously described by our laboratory and others [5], [13], [43], and may have a central role in pathogenesis of VILI [15], [44], [45]. In this study we identified MMP8 (neutrophil collagenase), an extracellular matrix-related protein, previously unknown to be involved in VILI. Winkler *et al.* found elevated MMP8 levels in endotracheal aspirate of children with respiratory distress [46]. Owen *et al.* described that the membrane-bound form of this enzyme regulates neutrophil migration in lung tissue [47]. In their model, *mmp8^−/−^* mice displayed increased inflammation 24 hours following LPS injection. Tester *et al.* have recently shown that *mmp8^−/−^* mice have an impaired initial response to LPS challenge, but once neutrophil infiltration takes place in the lung, the effect is sustained [20]. We observed increased MMP8 gene and protein expression following mechanical ventilation in wild-type mice. MMP8 was localized primarily on the cell surface of mononuclear cells as previously suggested [20]. *Mmp8^−/−^* mice responded to lung injury with increased microvascular permeability and alveolar protein accumulation relative to wild-type mice suggesting increased alveolo-capillary permeability to protein. However differences in neutrophil infiltration and pulmonary edema were not seen (Figure 11). In a model of wound healing Gutiérrez-Fernández *et al.* found that *mmp8^−/−^*mice have delayed neutrophil granulocyte apoptosis [48]. They speculated that extended neutrophil presence enhances local damage. In our context the delayed presence of inflammatory cells could lead to alveolar injury and subsequent permeability changes. In the same model the authors describe that MMP8 forms complexes with MMP9. In the absence of MMP8, increased MMP9 production was detected. Elevated MMP9 levels lead to increased vascular permeability in a model of hyperoxia and endotoxin-induced lung injury [49]. The effect of increased MMP8 expression in our model is detailed in Figure 12. This new observation sheds light on a previously unknown mechanism in VILI, which warrants further investigation.

*Jnk1^−/−^* mice were relatively resistant to VILI, and their global gene expression patterns differed from that of wild-type mice. Besides their gray color (versus black for wild type and *mkk3^−/−^* mice), *jnk1^−/−^* mice do not differ phenotypically from their wild-type counterparts. We speculated that analysis of the gene expression profile in *jnk1^−/−^* mice would provide information on their resistance to injury. The *jnk1^−/−^* mice responded to ventilation with the downregulation of genes significantly overrepresented in the immune response system (eg., Ccl8, Il1) and biopolymer metabolic processes (e.g., Mmp11) functional groups. A small number of genes enriched in acute phase response functional group were upregulated (e.g., Stat3). We did not observe a significant expression change of groups of inflammatory genes in *jnk1^−/−^* mice, which was prominent in ventilated wild-type mice (Figure 7A and C).

*Jnk1^−/−^* animals also displayed significant baseline overexpression of cytoskeleton genes and many were significantly enriched in the muscle contraction functional group (Figure 7B and C, Table S3). Cytoskeletal reorganization upon cyclic stretch has been demonstrated in pulmonary arterial endothelial cells [38]. Birukov *et al.* showed that cytoskeletal rearrangement depends on the magnitude of cell stretch. Other studies suggest that JNK inhibition attenuates actin cytoskeleton remodeling [50]. Our gene expression analysis confirms previous observations that JNK is involved both in the mechanotransducive and an inflammatory mechanism of VILI [31], [42], [51].

Increased alveolar epithelial cellular apoptosis has been noted following moderate tidal volume ventilation [52]. JNK signaling is pro-apoptotic and thus *jnk1* ^−/−^ mice are resistant to various pro-apoptotic stimuli [40]. We show that GADD45α expression in response to ventilation was further enhanced in the absence of JNK1. GADD45α has previously been identified as a candidate gene in VILI [5], [43]. GADD45α have been shown to regulate JNK and p38 MAPK signaling to promote apoptosis [53]. However others suggest that JNK activation can precede or occur independently of GADD45α activation [54]. At present, we can only speculate that JNK modulates GADD45α activation directly (Figure 12) or that the effect is mediated via nuclear-factor κB, a major regulator of both JNK and GADD45 [41].

We conclude that MAPK (including MKK3/p38 MAPK and JNK pathways) play a central role in coordinating various forms of injury displayed in VILI. MAPK are involved in transmitting signals of cellular inflammation, mechanotransduction, and apoptosis. A separate mechanism that involves MMP8 is responsible at least in part for increased alveolo-capillary protein permeability in VILI. Our results suggest that strategies to modulate MAPK and/or MMP8 activation may have potential therapeutic benefit in patients suffering from ventilator-associated lung injury/ARDS.

## Supporting Information

Table S1 Indices of lung injury in bronchoalveolar lavage fluid. wt = wild-type mouse,mkk3 = *mkk3^−/−^* mouse, jnk1 = *jnk1^−/−^* mouse, n = 5–8mice/group
(0.68 MB TIF)

Table S2 Mean blood pressure during 8 hours mechanical ventilation in wild-type mice. a = significant change 0^th^ versus 8^th^ hour ventilation, p<0.05, n = 3/group, abbreviation:BP = blood pressure
(0.12 MB TIF)

Table S3 103 genes with significant expression change following mechanical ventilation in wild-type and *jnk1^−/−^* mice. ^a^ wt = gene expression ratio wild-type mouse ventilation/control, *jnk1^−/−^* = gene expression ratio *jnk1^−/−^* mouse ventilation/control. Genes are ranked by fold-change. Listed genes meet the following statistical criteria significant change: t-tests p-value<0.05 with 5%FDR correction, TNoM score = 0 and fold-change of expression >2. The expression changes of genes in bolded font were confirmed with RT-PCR.
(0.07 MB DOC)

Figure S1 Time course of mechanical ventilation in wild-type, *mkk3^−/−^*, *jnk1^−/−^* and in *mmp8^−/−^* mice. Mice were ventilated with 10 or 20 ml/kg tidal volume and their lung injury parameters were compared to non-ventilated controls. *20 ml/kg tidal volume ventilation experiments for wild-type, mkk3^−/−^ and jnk1^−/−^ mice:* Mice were ventilated for 2 hours to assess changes in broncholveolar lavage fluid (BALF) total protein, total and differential cell count and tumor necrosis factor-μ levels (n = 5/group). Mice were ventilated for 4 hours to assess changes in BALF total protein, total and differential cell count. Lung tissue was harvested for protein expression analysis (n = 5/group). In a separate set of experiments microvascular permeability was measured in mice ventilated for 4 hours using Evans Blue (EB) dye extravasation method. Mice were injected with EB dye 2 hours prior the end of mechanical ventilation. At the end of the ventilation period left lung tissue and blood was collected for permeability measurement (n = 5/group). Wet-to-dry lung weight ratio was also measured by comparing the wet and desiccated weight of right lungs. *10 ml/kg tidal volume ventilation experiment for wild-type, jnk1^−/−^, mkk3^−/−^ and mmp8^−/−^ mice:* Mice were ventilated for 8 hours (n = 5/group) to assess changes in BALF (total protein, total and differential cell count) and in lung histology. Right lungs were used for BALF assessment and left lungs for histology and TUNEL staining. Additional lung tissue from separate experiments with wild-type and *jnk1^−/−^* mice was harvested for gene and protein expression, immunohistochemistry (n = 4/group). In a separate set of experiments microvascular permeability was measured in wild-type and *mmp8^−/−^* mice ventilated for 8 hours using EB dye extravasation method. Mice were injected with EB dye 2 hours prior the end of mechanical ventilation. At the end of the ventilation period lung tissue and blood was collected for permeability measurement (n = 3/group). We used 5–8 non-ventilated control animals/group to obtain baseline BALF, permeability and tissue parameters. abbreviation: BAL = bronchoalveolar lavage
(0.27 MB TIF)

## References

[1] Grasso S, Mascia L, Del Turco M, Malacarne P, Giunta F (2002). Effects of recruiting maneuvers in patients with acute respiratory distress syndrome ventilated with protective ventilatory strategy.. Anesthesiology.

[2] The ARDS Network (2000). Ventilation with lower tidal volumes as compared with traditional tidal volumes for acute lung injury and acute respiratory distress syndrome.. N Engl J Med.

[3] Brower RG, Lanken PN, MacIntyre N, Matthay MA, Morris A (2004). Higher versus lower positive end-expiratory pressures in patients with the acute respiratory distress syndrome.. N Engl J Med.

[4] Mayr VD, Dunser MW, Greil V, Jochberger S, Luckner G (2006). Causes of death and determinants of outcome in critically ill patients.. Crit Care.

[5] Altemeier WA, Matute-Bello G, Gharib SA, Glenny RW, Martin TR (2005). Modulation of lipopolysaccharide-induced gene transcription and promotion of lung injury by mechanical ventilation.. J Immunol.

[6] Bregeon F, Roch A, Delpierre S, Ghiago E, Autillo-Touati A (2002). Conventional mechanical ventilation of healthy lungs induced pro-inflammatory cytokine gene trascription.. Resp Physiol.

[7] Yang KY, Arcaroli JJ, Abraham E (2003). Early alterations in neutrophil activation are associated with outcome in acute lung injury.. Am J Respir Crit Care Med.

[8] Chess PR, O'Reilly MA, Sachs F, Finkelstein JN (2005). Reactive oxidant and p42/44 MAP kinase signaling is necessary for mechanical strain-induced proliferation in pulmonary epithelial cells.. J Appl Physiol.

[9] Abdulnour RE, Peng X, Finigan JH, Han EJ, Hasan EJ (2006). Mechanical stress activates xanthine oxidoreductase through MAP kinase-dependent pathways.. Am J Physiol Lung Cell Mol Physiol.

[10] Otterbein LE, Otterbein SL, Ifedigbo E, Liu F, Morse DE (2003). MKK3 mitogen-activated protein kinase pathway mediates carbon monoxide-induced protection against oxidant-induced lung injury.. Am J Pathol.

[11] Morse D, Otterbein LE, Watkins S, Alber S, Zhou Z (2003). Deficiency in the c-Jun NH2-terminal kinase signaling pathway confers susceptibility to hyperoxic lung injury in mice.. Am J Physiol Lung Cell Mol Physiol.

[12] Grigoryev DN, Ma SF, Irizarry RA, Ye SQ, Quackenbush J (2004). Orthologous gene-expression profiling in multi-species models: search for candidate genes.. Genome Biol.

[13] Dolinay T, Kaminski N, Felgendreher M, Kim HP, Reynolds P (2006). Gene expression profiling of target genes in ventilator-induced lung injury.. Physiol Genomics.

[14] Moitra J, Sammani S, Garcia JG (2007). Re-evaluation of Evans Blue dye as a marker of albumin clearance in murine models of acute lung injury.. Transl Res.

[15] Belperio JA, Keane MP, Burdick MD, Londhe V, Xue YY (2002). Critical role for CXCR2 and CXCR2 ligands during the pathogenesis of ventilator-induced lung injury.. J Clin Invest.

[16] Hales CA, Du HK, Volokhov A, Mourfarrej R, Quinn DA (2001). Aquaporin channels may modulate ventilator-induced lung injury.. Respir Physiol.

[17] Tremblay L, Valenza F, Riberio SP, Li J, Slutsky AS (1997). Injurious ventilatory strategies increase cytokines and c-fos m-RNA expression in an isolated lung rat model.. J Clin Invest.

[18] Otterbein LE, Bach FH, Alam J, Soares M, Tao Lu H (2000). Carbon monoxide has anti-inflammatory effects involving the mitogen-activated protein kinase pathway.. Nat Med.

[19] Morse D, Pischke SE, Zhou Z, Davis RJ, Flavell RA (2003). Suppression of inflammatory cytokine production by carbon monoxide involves the JNK pathway and AP-1.. J Biol Chem.

[20] Tester AM, Cox JH, Connor AR, Starr AE, Dean RA (2007). LPS Responsiveness and Neutrophil Chemotaxis In Vivo Require PMN MMP-8 Activity.. PLoS ONE.

[21] Fayolle C, Pourchet J, de Fromentel CC, Puisieux A, Dore JF (2008). Gadd45a activation protects melanoma cells from ultraviolet B-induced apoptosis.. J Invest Dermatol.

[22] Ning W, Chu TJ, Li CJ, Choi AM, Peters DG (2004). Genome-wide analysis of the endothelial transcriptome under short-term chronic hypoxia.. Physiol Genomics.

[23] Wu W, Dave N, Tseng GC, Ricards T, Xing EP (2005). Comparison of normalization methods for CodeLink Bioarray data.. BMC Bioinformatics.

[24] Benjamini Y, Hochberg Y (1995). Controlling the false discovery rate: A practical and powerful approach to multiple testing.. J Roy Statist Soc Ser B.

[25] Tusher VG, Tibshirani R, Chu G (2001). Significance analysis of microarrays applied to ionizing radiation response.. Proc Natl Acad Sci U S A.

[26] Song R, Kubo M, Morse D, Zhou Z, Zhang X (2003). Carbon monoxide induces cytoprotection in rat orthotopic lung transplantation via anti-inflammatory and anti-apoptotic effects.. Am J Pathol.

[27] Gavrieli Y, Sherman Y, Ben-Sasson SA (1992). Identification of programmed cell death in situ via specific labeling of nuclear DNA fragmentation.. J Cell Biol.

[28] von Bethmann AN, Brasch F, Nusing R, Vogt K, Volk HD (1998). Hyperventilation induces release of cytokines from perfused mouse lung.. Am J Respir Crit Care Med.

[29] Matthay MA, Bhattacharya S, Gaver D, Ware LB, Lim LH (2002). Ventilator-induced lung injury: in vivo and in vitro mechanisms.. Am J Physiol Lung Cell Mol Physiol.

[30] Uhlig U, Haitsma JJ, Goldmann T, Poelma DL, Lachmann B (2002). Ventilation-induced activation of the mitogen-activated protein kinase pathway.. Eur Respir J.

[31] Li LF, Yu L, Quinn DA (2004). Ventilation-induced neutrophil infiltration depends on c-Jun N-terminal kinase.. Am J Respir Crit Care Med.

[32] de Prost N, Dreyfuss D, Saumon G (2007). Evaluation of two-way protein fluxes across the alveolo-capillary membrane by scintigraphy in rats: effect of lung inflation.. J Appl Physiol.

[33] Quinn DA, Moufarrej RK, Volokhov A, Hales CA (2002). Interactions of lung stretch, hyperoxia, and MIP-2 production in ventilator-induced lung injury.. J Appl Physiol.

[34] Petrache I, Birukova A, Ramirez SI, Garcia JG, Verin AD (2003). The role of the microtubules in tumor necrosis factor-alpha-induced endothelial cell permeability.. Am J Respir Cell Mol Biol.

[35] Crimi E, Zhang H, Han RN, Sorbo LD, Ranieri VM (2006). Ischemia and reperfusion increases susceptibility to ventilator-induced lung injury in rats.. Am J Respir Crit Care Med.

[36] Matute-Bello G, Liles WC, Steinberg KP, Kiener PA, Mongovin S (1999). Soluble Fas ligand induces epithelial cell apoptosis in humans with acute lung injury (ARDS).. J Immunol.

[37] Nakamura M, Matute-Bello G, Liles WC, Hayashi S, Kajikawa O (2004). Differential response of human lung epithelial cells to fas-induced apoptosis.. Am J Pathol.

[38] Birukov KG, Jacobson JR, Flores AA, Ye SQ, Birukova AA (2003). Magnitude-dependent regulation of pulmonary endothelial cell barrier function by cyclic stretch.. Am J Physiol Lung Cell Mol Physiol.

[39] Matute-Bello G, Liles WC, Radella F, Steinberg KP, Ruzinski JT (1997). Neutrophil apoptosis in the acute respiratory distress syndrome.. Am J Respir Crit Care Med.

[40] Davis RJ (2000). Signal transduction by the JNK group of MAP kinases.. Cell.

[41] Papa S, Zazzeroni F, Pham CG, Bubici C, Franzoso G (2004). Linking JNK signaling to NF-kappaB: a key to survival.. J Cell Sci.

[42] Li LF, Liao SK, Ko YS, Lee CH, Quinn DA (2007). Hyperoxia increases ventilator-induced lung injury via mitogen-activated protein kinases: a prospective, controlled animal experiment.. Crit Care.

[43] Ma SF, Grigoryev DN, Taylor AD, Nonas S, Sammani S (2005). Bioinformatic identification of novel early stress response genes in rodent models of lung injury.. Am J Physiol Lung Cell Mol Physiol.

[44] Tschumperlin DJ, Oswari J, Margulies AS (2000). Deformation-induced injury of alveolar epithelial cells. Effect of frequency, duration, and amplitude.. Am J Respir Crit Care Med.

[45] Jones JC, Lane K, Hopkinson SB, Lecuona E, Geiger RC (2005). Laminin-6 assembles into multimolecular fibrillar complexes with perlecan and participates in mechanical-signal transduction via a dystroglycan-dependent, integrin-independent mechanism.. J Cell Sci.

[46] Winkler MK, Foldes JK, Bunn RC, Fowlkes JL (2003). Implications for matrix metalloproteinases as modulators of pediatric lung disease.. Am J Physiol Lung Cell Mol Physiol.

[47] Owen CA, Hu Z, Lopez-Otin C, Shapiro SD (2004). Membrane-bound matrix metalloproteinase-8 on activated polmorphonuclear cells is a potent,tissue inhibitor of metalloproteinase-resistant collagenase and serpinase.. J Immunol.

[48] Gutierez-Fernandez A, Inada M, Balbin M, Fueyo A, Pitiot A (2007). Increased inflammation delays wound healing in mice deficient in collagenase-2 (MMP8).. Faseb J.

[49] Kohno K, Ishizaka A, Sawafuji M, Koh H, Hirayama Y (2004). Hyperoxia-induced emphysematous changes in subacute phase of endotoxin-induced lung injury in rats.. Am J Physiol Lung Cell Mol Physiol.

[50] Usatyuk PV, Natarajan V (2003). Role of Mitogen-activated Protein Kinases in 4-Hydroxy-2-nonenal-induced Actin Remodeling and Barrier Function in Endothelial Cells.. J Biol Chem.

[51] Oudin S, Pugin J (2002). Role of MAP kinase activation in interleukin-8 production by human BEAS-2B bronchial epithelial cells submitted to cyclic stretch.. Am J Respir Cell Mol Biol.

[52] Imai Y, Parodo J, Kajikawa O, de Perrot M, Fischer S (2003). Injurious mechanical ventilation and end-organ epithelial cell apoptosis and organ dysfunction in an experimental model of acute respiratory distress syndrome.. Jama.

[53] Zerbini L, Wang Y, Czibere A, Correa R, Cho J (2004). Nf-kB-mediated repression of growth arrest- and DNA demage-inducible alpha and gamma is essential for cancer cell survival.. PNAS.

[54] Shaulian E, Karin M (1999). Stress-induced JNK activation in independnet of GAD45 induction.. J Biol Chem.

