# Supplementary figures and images for: Mitogen-Activated Protein Kinases Regulate Susceptibility to Ventilator-Induced Lung Injury
Source: PLoS One. 2008 Feb 13;3(2):e1601. doi: 10.1371/journal.pone.0001601 (PMC2223071; doi:10.1371/journal.pone.0001601)

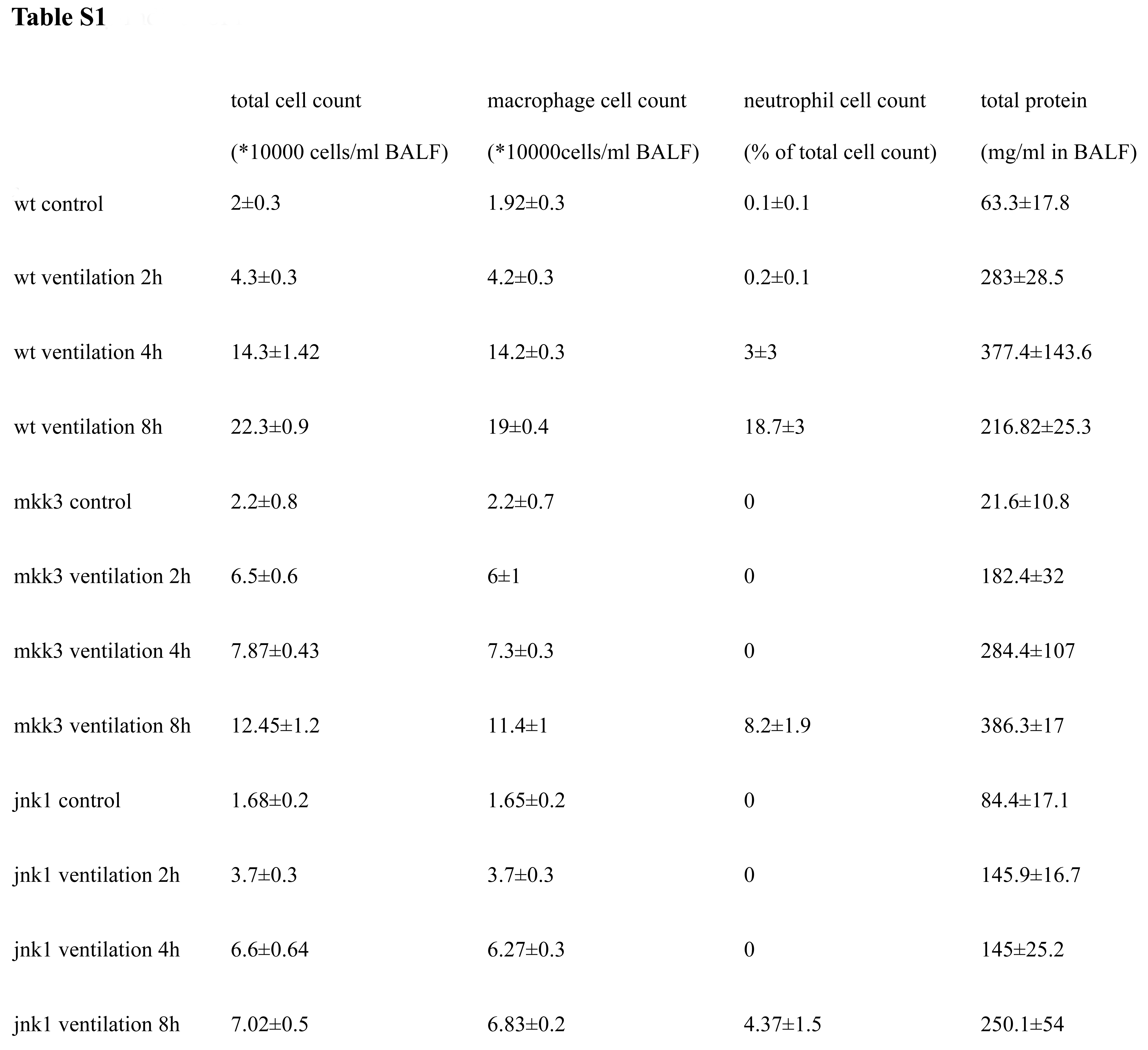

Supplement: Table S1 — Indices of lung injury in bronchoalveolar lavage fluid. wt = wild-type mouse,mkk3 = mkk3−/− mouse, jnk1 = jnk1−/− mouse, n = 5–8mice/group (0.68 MB TIF) [file pone.0001601.s001.tif]

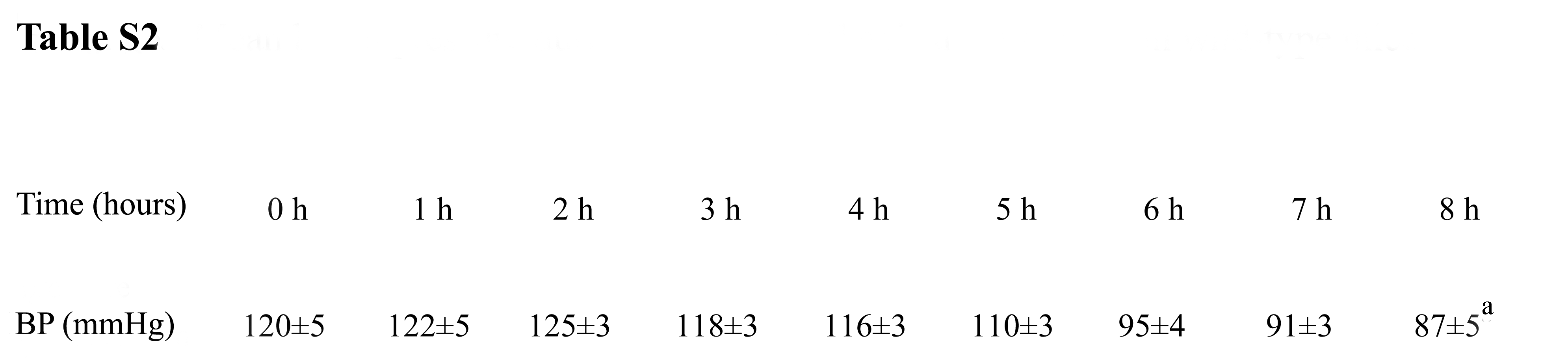

Supplement: Table S2 — Mean blood pressure during 8 hours mechanical ventilation in wild-type mice. a = significant change 0th versus 8th hour ventilation, p<0.05, n = 3/group, abbreviation:BP = blood pressure (0.12 MB TIF) [file pone.0001601.s002.tif]

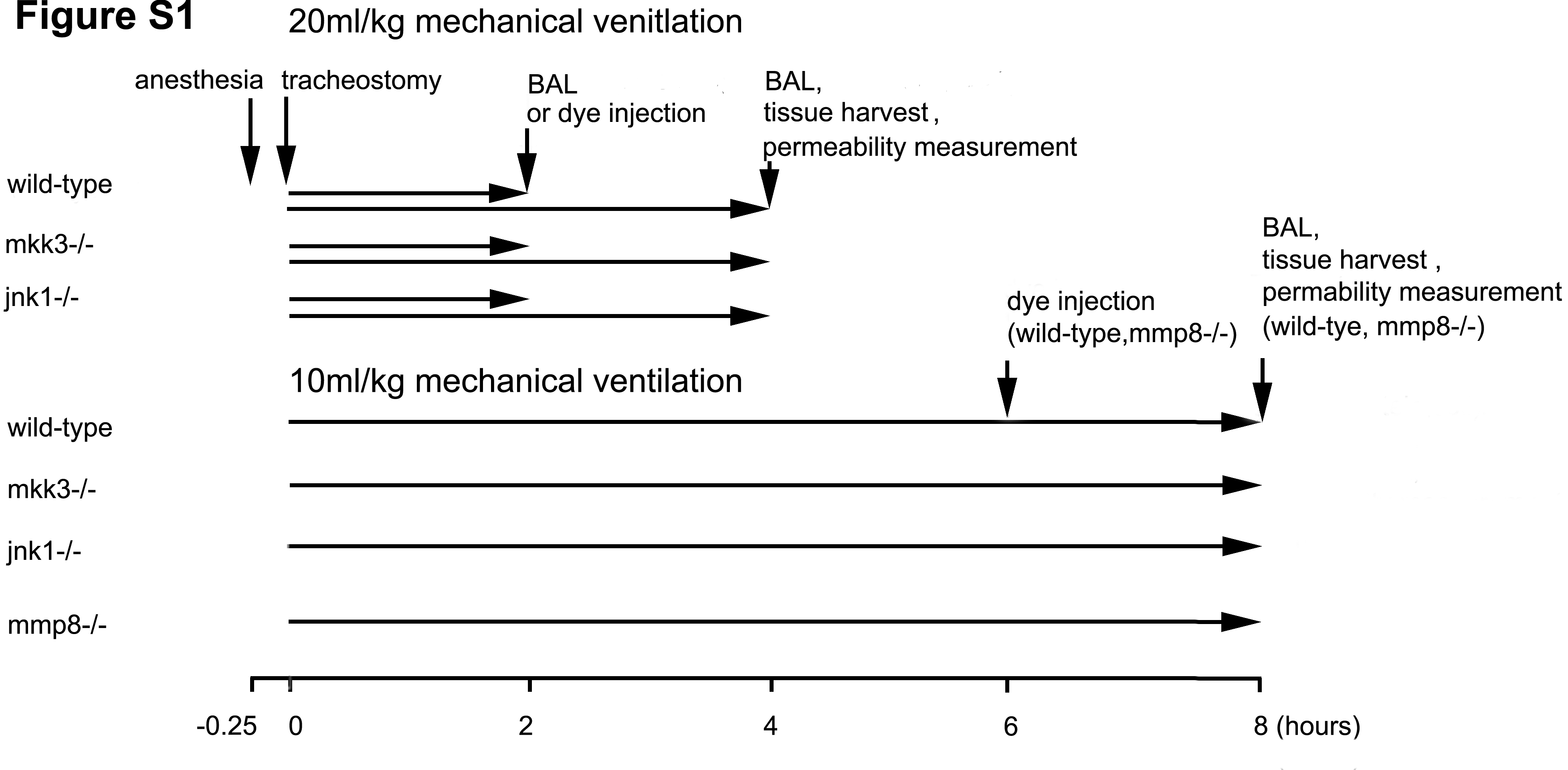

Supplement: Figure S1 — Time course of mechanical ventilation in wild-type, mkk3−/−, jnk1−/− and in mmp8−/− mice. Mice were ventilated with 10 or 20 ml/kg tidal volume and their lung injury parameters were compared to non-ventilated controls. 20 ml/kg tidal volume ventilation experiments for wild-type, mkk3−/− and jnk1−/− mice: Mice were ventilated for 2 hours to assess changes in broncholveolar lavage fluid (BALF) total protein, total and differential cell count and tumor necrosis factor-μ levels (n = 5/group). Mice were ventilated for 4 hours to assess changes in BALF total protein, total and differential cell count. Lung tissue was harvested for protein expression analysis (n = 5/group). In a separate set of experiments microvascular permeability was measured in mice ventilated for 4 hours using Evans Blue (EB) dye extravasation method. Mice were injected with EB dye 2 hours prior the end of mechanical ventilation. At the end of the ventilation period left lung tissue and blood was collected for permeability measurement (n = 5/group). Wet-to-dry lung weight ratio was also measured by comparing the wet and desiccated weight of right lungs. 10 ml/kg tidal volume ventilation experiment for wild-type, jnk1−/−, mkk3−/− and mmp8−/− mice: Mice were ventilated for 8 hours (n = 5/group) to assess changes in BALF (total protein, total and differential cell count) and in lung histology. Right lungs were used for BALF assessment and left lungs for histology and TUNEL staining. Additional lung tissue from separate experiments with wild-type and jnk1−/− mice was harvested for gene and protein expression, immunohistochemistry (n = 4/group). In a separate set of experiments microvascular permeability was measured in wild-type and mmp8−/− mice ventilated for 8 hours using EB dye extravasation method. Mice were injected with EB dye 2 hours prior the end of mechanical ventilation. At the end of the ventilation period lung tissue and blood was collected for permeability measurement (n = 3/group). We used 5 [file pone.0001601.s004.tif]
