# Supplementary material for: Mitogen-Activated Protein Kinases Regulate Susceptibility to Ventilator-Induced Lung Injury
Source: PLoS One. 2008 Feb 13;3(2):e1601. doi: 10.1371/journal.pone.0001601 (PMC2223071; doi:10.1371/journal.pone.0001601)
Supplement: Table S3 — 103 genes with significant expression change following mechanical ventilation in wild-type and jnk1−/− mice. a wt = gene expression ratio wild-type mouse ventilation/control, jnk1−/− = gene expression ratio jnk1−/− mouse ventilation/control. Genes are ranked by fold-change. Listed genes meet the following statistical criteria significant change: t-tests p-value<0.05 with 5%FDR correction, TNoM score = 0 and fold-change of expression >2. The expression changes of genes in bolded font were confirmed with RT-PCR. (0.07 MB DOC) [file pone.0001601.s003.doc]

**Table S3**

| **Genes regulated in wild-type and in *jnk1-/-*mice** | | | |
| --- | --- | --- | --- |
| ***Uniene ID*** | ***Gene Name*** | ***^a^wt*** | ***jnk1-/-*** |
| **Mm.16415** | **matrix metalloproteinase 8 (Mmp8)** | **40.06** | **43.21** |
| Mm.142740 | metallothionein-2 (MT2) | 16.37 | 13.43 |
| Mm.174615 | cDNA sequence BC019943 (BC019943) | 10.67 | 8.60 |
| Mm.24920 | macrophage C-type lectin (Mpcl) | 10.17 | 11.32 |
| Mm.38169 | RIKEN clone:3110001I22 product:weakly similar to apoptosis regulator | 8.74 | 9.10 |
| Mm.36640 | mitogen-activated protein kinase kinase kinase 6 (Map3k6) | 7.68 | 9.71 |
| Mm.4368 | glutamate-cysteine ligase catalytic subunit (GLCLC) | 7.23 | 6.36 |
| Mm.262670 | serine protease inhibitor-2 related sequence 1 (Spi2-rs1) | 6.78 | 3.81 |
| Mm.34242 | RIKEN cDNA 1110011E12 gene (1110011E12Rik) | 5.40 | 7.98 |
| Mm.22903 | polymeric immunoglobulin receptor 3 precursor (Pigr3) | 5.29 | 4.08 |
| Mm.159762 | adult male pituitary gland cDNA RIKEN clone:5330417H12 product | 5.18 | 5.69 |
| Mm.28518 | tumor necrosis factor receptor superfamily member 12a (Tnfrsf12a) | 5.00 | 7.74 |
| Mm.14191 | kallikrein binding protein (Klkbp) | 4.85 | 3.09 |
| Mm.233802 | interleukin 4 receptor alpha (Il4ra) | 4.64 | 3.60 |
| Mm.6215 | fos-like antigen 1 (Fosl1) | 4.62 | 4.41 |
| Mm.9653 | growth arrest and DNA-damage-inducible 45 gamma (Gadd45g) | 4.39 | 3.72 |
| Mm.1408 | adrenomedullin (Adm) | 4.37 | 6.31 |
| Mm.249379 | interleukin 1 family member 9 (Il1f9) | 4.16 | 3.44 |
| Mm.15801 | lipocalin 7 (Lcn7) | 4.03 | 4.87 |
| Mm.19788 | basic transcription element binding protein 1 (Bteb1) | 3.98 | 3.52 |
| Mm.150899 | UI-M-BH4-azn-d-08-0-UI.s1 NIH_BMAP_M_S5 cDNA clone | 3.61 | 4.36 |
| Mm.10948 | schlafen 1 (Slfn1) | 3.57 | 4.88 |
| Mm.45815 | breast cancer anti-estrogen resistance 3 (Bcar3) | 3.45 | 4.96 |
| Mm.251657 | TBC1 domain family member 8 (Tbc1d8) | 3.11 | 4.42 |
| Mm.255080 | DNA segment Chr 7 ERATO Doi 458 expressed (D7Ertd458e) | 2.81 | 3.10 |
| Mm.5079 | hydroxysteroid 11-beta dehydrogenase 2 (Hsd11b2) | 2.72 | 2.39 |
| Mm.7446 | RIKEN cDNA 8430417G17 gene (8430417G17Rik) | 2.65 | 4.40 |
| Mm.33709 | CEA-related cell adhesion molecule 14 (Ceacam14) | 2.55 | 2.41 |
| Mm.200426 | LIM and cysteine-rich domains 1 (Lmcd1) | 2.49 | 3.02 |
| Mm.176347 | 13 days embryo stomach cDNA RIKEN clone:D530033C11 | 2.46 | 2.42 |
| Mm.60997 | hypothetical protein 4932411E22 (4932411E22) | 2.40 | 2.39 |
| Mm.257667 | RIKEN clone:2700050C19 | 2.23 | 3.18 |
| **Mm.1236** | **growth arrest and DNA-damage-inducible 45 alpha (Gadd45a)** | **2.15** | **4.16** |
| Mm.168110 | RIKEN clone:5330403D14 | 2.01 | 5.77 |
| Mm.29813 | RIKEN clone:1700029G01 | 1.97 | 4.47 |
| Mm.87858 | bone gamma carboxyglutamate protein 1 (Bglap1) | 0.99 | 0.44 |
| Mm.82624 | RIKEN cDNA E430021N18 gene (E430021N18Rik) | 0.83 | 0.43 |
| Mm.22049 | cysteine rich intestinal protein (Crip) | 0.49 | 0.23 |
| Mm.241656 | monoamine oxidase B (Maob) | 0.48 | 0.49 |
| Mm.14526 | myosin light chain, fast skeletal muscle (Mylpf) | 0.47 | 0.01 |
| Mm.46172 | cAMP-dependent protein kinase regulatory subunit | 0.43 | 0.37 |
| Mm.41279 | RIKEN clone:6620401M08 | 0.43 | 0.44 |
| Mm.268037 | RIKEN clone:1700019L03 | 0.43 | 0.26 |
| Mm.46759 | cDNA sequence BC027340 (BC027340) | 0.43 | 0.46 |
| Mm.12863 | heparan sulfate 2-O-sulfotransferase 1 (Hs2st1) | 0.40 | 0.40 |
| Mm.33810 | RIKEN clone:1700010A17 | 0.39 | 0.42 |

**Genes regulated in wild-type and in *jnk1-/-*mice (continued)**

| ***Uniene ID*** | ***Gene Name*** | ***wt*** | ***jnk1-/-*** |
| --- | --- | --- | --- |
| Mm.269989 | RIKEN cDNA 2810046M22 gene (2810046M22Rik) | 0.36 | 0.48 |
| Mm.100671 | ATP-binding cassette sub-family G (WHITE) member 3 (Abcg3) | 0.36 | 0.38 |
| Mm.23991 | RIKEN cDNA 5830443L24 gene (5830443L24Rik) | 0.32 | 0.37 |
| Mm.10747 | allograft inflammatory factor 1 (Aif1) | 0.30 | 0.14 |
| Mm.24118 | glutathione S-transferase theta 2 (Gstt2) | 0.29 | 0.22 |
| Mm.154109 | RIKEN clone:1810009F10 product:weakly similar to MSTP014 | 0.25 | 0.41 |
| Mm.102196 | RIKEN clone:2900001G08 | 0.24 | 0.14 |
| Mm.32411 | immune associated nucleotide 3 (Ian3) | 0.22 | 0.35 |
| Mm.17510 | mesothelin (Msln) | 0.21 | 0.42 |
| Mm.33613 | clone IMAGE:1380300 partial cds | 0.20 | 0.22 |
| Mm.74335 | hypothetical protein B230342N19 (B230342N19) | 0.20 | 0.44 |
| Mm.27579 | RIKEN clone:2900062L11 product:similar to microsomal signal peptidase | 0.18 | 0.29 |
| Mm.878 | lymphocyte antigen 6 complex, locus D (Ly6d) | 0.17 | 0.12 |
| **Mm.13828** | **WNT1 inducible signaling pathway protein 2 (Wisp2)** | **0.16** | **0.35** |

| **Genes regulated primarily in *jnk1-/-* mice** | | | |
| --- | --- | --- | --- |
| ***Uniene ID*** | ***Gene Name*** | ***wt*** | ***jnk1-/-*** |
| Mm.33172 | RIKEN clone:A930008K15 | 1.69 | 8.34 |
| Mm.21177 | RIKEN cDNA A930014I12 gene (A930014I12Rik) | 0.77 | 7.24 |
| Mm.3337 | selectin platelet (Selp) | 4.53 | 5.11 |
| Mm.1453 | a disintegrin and metalloproteinase domain 1a (Adam1a) | 1.13 | 4.88 |
| Mm.158737 | RIKEN clone:9130004J05 | 1.15 | 5.07 |
| Mm.41077 | RIKEN clone:2810432C06 | 1.56 | 4.11 |
| Mm.152250 | UI-M-BZ1-bjb-f-07-0-UI.s1 NIH_BMAP_MHI2_S1 cDNA clone | 0.86 | 3.57 |
| Mm.5262 | choline kinase (Chk) | 1.55 | 3.27 |
| **Mm.641** | **activating transcription factor 4 (Atf4)** | **1.56** | **3.15** |
| Mm.77743 | H3001E08-3 NIA Mouse 15K cDNA Clone | 1.12 | 2.86 |
| Mm.30059 | myristoylated alanine rich protein kinase C substrate (Marcks) | 1.38 | 2.83 |
| Mm.22704 | adenylate cyclase inhibitory G protein i3 alpha chain {clone G13} | 1.95 | 2.61 |
| Mm.7221 | low density lipoprotein receptor-related protein 1 (Lrp1) | 1.71 | 2.60 |
| Mm.30144 | cytotoxic T lymphocyte-associated protein 2 alpha (Ctla2a) | 1.64 | 2.55 |
| Mm.28726 | nucleostemin (Ns-pending) transcript variant 1 | 1.48 | 2.25 |
| Mm.27932 | arsenate resistance protein 2 (Ars2-pending) | 1.29 | 2.14 |
| Mm.12829 | calsequestrin 1 (Casq1) | 1.00 | 0.17 |
| Mm.214950 | skeletal muscle actin | 1.21 | 0.16 |
| Mm.39469 | troponin I skeletal fast 2 (Tnni2) | 0.95 | 0.07 |
| Mm.21075 | calmodulin 4 (Calm4) | 1.10 | 0.05 |
| Mm.35134 | ATPase Ca++ transporting cardiac muscle fast twitch 1 (Atp2a1) | 0.97 | 0.05 |
| Mm.207067 | defensin beta 7 (Defb7) | 1.06 | 0.04 |
| **Genes regulated primarily in wild-type mice** | | | |
| Mm.100110 | RIKEN clone:9130015A21 | 2.61 | 0.78 |
| Mm.24750 | protein distantly related to to the gamma subunit family (Pr1) | 2.23 | 1.11 |
| Mm.2549 | hexokinase 2 (Hk2) | 2.18 | 1.78 |
| Mm.160094 | RIKEN clone:5430417C01 | 2.09 | 1.91 |
| Mm.44136 | RalBP1 associated Eps domain containing protein 2 (Reps2) | 2.07 | 0.81 |
| Mm.101159 | UI-M-BH3-aqx-h-02-0-UI.s1 NIH_BMAP_M_S4 cDNA clone | 0.48 | 0.51 |
| Mm.156884 | BC024400 (BC024400) | 0.47 | 0.56 |
| Mm.29431 | RIKEN cDNA 1810014L12 gene (1810014L12Rik) | 0.46 | 0.70 |
| Mm.32764 | cDNA clone 1700007L15 | 0.46 | 0.54 |
| Mm.81122 | RIKEN clone:1110027G09 | 0.45 | 0.62 |
| Mm.181061 | RIKEN cDNA 4921522K17 gene (4921522K17Rik) | 0.43 | 1.09 |
| Mm.22270 | transmembrane 4 superfamily member 3 (Tm4sf3) | 0.40 | 0.58 |
| Mm.136892 | hypothetical protein 8430408F21 (8430408F21) | 0.39 | 0.57 |
| Mm.29310 | RIKEN clone:5730419O14 product:Aldo/keto reductase family | 0.35 | 0.59 |
| Mm.158729 | RIKEN clone:9030622O22 | 0.34 | 0.61 |
| Mm.86627 | RIKEN cDNA 2210010B09 gene (2210010B09Rik) | 0.34 | 0.66 |
| Mm.20892 | RIKEN clone:2900082I03 product:synapsin II | 0.27 | 0.51 |
| Mm.94160 | brain expressed X-linked 2 (Bex2) | 0.20 | 0.66 |
